# High-Protein Diet Containing Dairy Products is Associated with Low Body Mass Index and Glucose Concentrations: A Cross-Sectional Study

**DOI:** 10.3390/nu11061384

**Published:** 2019-06-20

**Authors:** Bruna M. Giglio, Valeska I. R. Duarte, Ana Flávia Galvão, Ana Clara B. Marini, Raquel M. Schincaglia, João F. Mota, Luciana B. Souza, Gustavo D. Pimentel

**Affiliations:** Clinical and Sports Nutrition Research Laboratory (Labince), Faculty of Nutrition, Federal University of Goiás, Goiânia, Goiás 7605-080, Brazil; brunamgiglio@gmail.com (B.M.G.); valeskainques@gmail.com (V.I.R.D.); ana.galvao.1822@gmail.com (A.F.G.); ac.marini22@gmail.com (A.C.B.M.); raquelms@outlook.com (R.M.S.); jffmota@gmail.com (J.F.M.); lucianabronzi@ufg.com (L.B.S.)

**Keywords:** protein, dairy products, body mass index, obesity, blood glucose

## Abstract

The aim was to evaluate whether the ingestion of a high protein diet containing dairy protein is associated with anthropometric indicators of adiposity and blood glucose. Methods: A cross-sectional study was conducted with volunteers of 20–89 years of age who performed leisure physical activity. We assessed dietary intake, body mass index (BMI), waist circumference (WC), triceps skinfold thickness (TSFT), random blood glucose as well as sociodemographic and behavioral variables. Results: A total of 418 individuals were evaluated. The consumption of a high-protein diet (1.80 ± 0.49 g/kg/day) was found in 37.8% of individuals, which showed lower BMI, WC, TSFT and blood glucose concentrations compared to those with a low-protein diet (0.56 ± 0.18 g/kg/day). Dairy products consumption was inversely associated with BMI when adjusted for sex and age (odds ratio (OR): 0.79, 95% confidence interval (CI): 0.68–0.93, *p* = 0.004) and by sex, age, fiber and energy (OR: 0.79; 95% CI: 0.67–0.92; *p* = 0.004), and with blood glucose when adjusted for sex and age (OR: 0.86; 95% CI: 0.74–0.99; *p* = 0.042). Cheese consumption was inversely associated with blood glucose when adjusted for sex and age (OR: 0.73, 95% CI: 0.55–0.96, *p* = 0.023) and by sex, age, calories and fibers (OR: 0.74, 95% CI: 0.56–0.98, *p* = 0.036). Two portions of cheeses/day reduced the risk of having high blood glucose levels by approximately 80%. Conclusion: A high-protein diet containing dairy food, in particular two servings of cheese, was associated with low BMI and random glucose concentration.

## 1. Introduction

Despite the fact that overeating is related to obesity and type 2 diabetes mellitus (DM2) [1], it is suggested that protein intake above the recommended dietary intake (>1.2 g/kg/day) reduces body weight [2]. The probable mechanisms involved would be the increase of thermogenesis and satiety, which contribute to the energy balance [3,4].

Protein can be derived from animal and plant sources. Plant-based proteins can delay gastric emptying, helping to maintain body weight [5]. However, the benefits may be related not only to the proteins that are present in these foods but also to fibers that promote satiety [3,6]. In addition, the plant-based protein has low biological value, since it contains lower digestibility, essential amino acids and branched chain amino acids (BCAA). In contrast, protein-rich foods of animal origin (meat, eggs, and chickens) are considered have high biological value [2].

Among the sources of animal protein, dairy products such as milk, yogurt, and cheese can increase lean mass, while decreasing fat mass and the risk of DM2 [7]. These benefits are derived not only from protein but also from calcium, which improves β-pancreatic cells function, reduces lipogenesis, increases lipolysis and contributes to decreased body adiposity [8]. Another benefit is the amino acid profile of dairy products. Immunoglobulins, α-lactoglobulin, β-lactalbumin, albumin, and caseins, besides helping to maintain muscle mass, also reduce glycemia, appetite (by increasing anorectic hormones) and consequently adiposity [2,8]. 

However, few studies have investigated the associations between different sources of protein with anthropometric indicators as well as the random blood glucose. Therefore, the aim of this study was to evaluate whether the ingestion of a high-protein diet (above 1.2 g/kg/day) containing dairy protein are associated with anthropometric indicators of adiposity and blood glucose in leisure physical activity practitioners.

## 2. Materials and Methods 

### 2.1. Design of Study 

A cross-sectional study was conducted from May to July 2016 with participants recruited in five different public parks in Goiânia, GO. The inclusion criteria were men and women adults (20–59 years old) and elderly (≥60 years old) who attended in the parks to carry out leisure physical activities. Exclusion criteria included individuals with mobility impairment that limited the practice of physical activity. 

All participants signed the informed consent form designed according to the n° 466/12 on “Research involving human beings, from the Health Board of the Ministry of Health”. The Research Ethics Committee of the Federal University of Goiás, protocol number 1.470.285, approved the study.

### 2.2. Physical Activity

The data were collected by trained interviewers who asked to the participants: “Do you practice physical activity? What type of activity and frequency?” Physical activity was estimated according to World Health Organization (WHO) recommendations for adults and older adults [9]. Regular physical activity individuals were defined as individuals who had at least 150 min of moderate-intensity aerobic physical activity throughout the week. 

### 2.3. Anthropometric Measurements

Body weight was measured in a digital scale of the brand Filizola^®^ (São Paulo, Brazil) with a precision of 0.1 kg and height by a portable stadiometer (SECA^®^, Hamburg, Germany) with precision in millimeters for calculation of the body mass index (BMI), considering an overweight BMI to be ≥25 kg/m^2^ for adults and ≥27 kg/m^2^ for the elderly [1,10]. Measurements of body weight and height were performed according to the procedures described by Lohman et al. [11].

Abdominal obesity was determined by waist circumference (WC) using an inelastic tape with precision in millimeters. It was considered a high risk indicator for cardiovascular diseases to have a WC ≥80 cm for women and ≥94 cm for men [12]. The triceps skinfold thickness (TSFT) was measured in triplicate at the midpoint of the upper arm using the Lange^®^ skinfold (Santa Cruz, CA, USA) and was classified as high when the percentile according to age was ≥90 as proposed by Frisancho [13]. BMI and WC were measured only once in the study.

### 2.4. Random Glucose Assessment

Finger samples were collected only once using either side of the tip of middle finger on either hand and random glucose was measured using the portable monitor and reagent strips (Roche^®^, Mannheim, Germany). The results were evaluated and considered to be elevated when random blood glucose was ≥200 mg/dL according to the recommendations of the Standard Medical Care in Diabetes [14].

### 2.5. Food Intake

The usual food recall was evaluated based on a 24-h dietary recall by trained interviewers to obtain the total calories, macronutrients, protein sources, and fiber intake [15]. The nutritional analysis of the usual food recall was performed by Dietpro^®^ software (version 5.8, Minas Gerais, Viçosa, Brazil) using foods from the American Food Composition Table (United States Department of Agriculture) [16].

In order to obtain the protein sources, we determined the amount of protein portions that each participant consumed of the following groups: dairy products (milk, cheese, curd cheese, curdled milk, skimmed yogurt and whole yogurt), legumes (plant-based sources of protein: pea, bean, chickpeas, lentils and soy), meat (bovine, poultry, pork and fish), and eggs. Portions were defined according to the Food Guide for the Brazilian Population [17]: milk (182 g), cheese (50 g), curd cheese (45 g), curdled milk (77 g), skimmed yogurt (330 g), whole yogurt (165 g), legumes (plant-based sources of protein: pea, bean, chickpeas, lentils and soy) (86 g), beef (64 g), pork (93.5 g), poultry (100 g), fish (100 g) and eggs (50 g).

The cut-off values of Recommended Dietary Allowance (RDA) were used to define the low-protein (<0.8 g/kg/day), normoprotein (0.8–1.2 g/kg/day) and high-protein diet (>1.2 g/kg/day).

### 2.6. Statistical Analysis

Data were described as means and standard deviations or absolute and relative frequencies. Normality was assessed with the Shapiro-Wilk test. The comparisons of means between groups were performed using one-way ANOVA for variables with normal distribution or by a Kruskal-Wallis test without normality. The chi-square test was performed for the categorical variables test.

Logistic regression was used to calculate the odds ratio (95%-CI) in order to evaluate the association of anthropometric measures, random blood glucose (main outcome) and the consumption of portions/protein groups and total protein intake (independent variables). The regression analysis logistic were performed unadjusted (crude model); adjusted for age and sex (model 1) and adjusted for age, sex, calories, and dietary fiber intake (model 2). The statistical significance level was set at *p* < 0.05. All analysis was performed using STATA (version 12, College Station, TX, USA).

## 3. Results

### 3.1. Subject Characteristics

The sociodemographic variables are described in Table 1. A total of 418 participants were evaluated, comprising 75% adults (median, age 38 years of age (20–59)), 25% who were elderly (median, age 65 year (60–89)) and 33% who practiced regular physical activity. Participants consuming a high-protein diet (37.8%) also showed higher intake of calories, lipids, and fiber when compared to those with a low-protein diet. No differences were observed in sex, race, smoking habits, and alcoholic beverages. In addition, the high protein diet group presented lower BMI, WC, TSFT, and concentrations of random blood glucose compared to the low-protein diet group (Table 1). 

### 3.2. Association Between Legume Protein with Anthropometric Measures and Blood Glucose 

The consumption of legume protein was not associated with anthropometric measures and random blood glucose (*p* > 0.05) (Table 2). 

### 3.3. Association between Total Protein Intake with Anthropometric Measures and Blood Glucose 

The higher protein intake was associated with reduced odds of BMI classified as overweight in model 1 (OR: 0.47, 95%-CI: 0.33–0.67, *p* < 0.001) and in model 2 (OR: 0.26, 95%-CI: 0.16–0.44, *p* < 0.001); TSFT classified as high in model 1 (OR: 0.52, 95%-CI: 0.34–0.79, *p* = 0.002) and in model 2 (OR: 0.27, 95%-CI: 0.15–0.51, *p* < 0.001) as well as WC classified as high in model 1 (OR: 0.44, 95%-CI: 0.30–0.65, *p* < 0.001) and in model 2 (OR: 0.25, 95%-CI: 0.14–0.43, *p* < 0.001). Additionally, the consumption of protein was associated with a lower glucose level in model 1 (OR: 0.60, 95% CI: 0.42–0.86, *p* = 0.006) and in model 2 (OR: 0.56, 95%-CI: 0.34–0.91, *p* = 0.019) (Table 3).

### 3.4. Association between Dairy Intake with Anthropometrics Measurements and Blood Glucose

**Figure 1:**
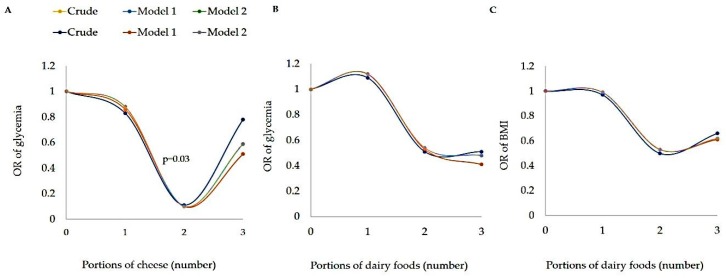
Association between glucose and portions of cheese **(A)**, glucose and portions of dairy food (**B)** and BMI and dairy food **(C)**—BMI: body mass index. Logistic regression analysis with OR (Odds Ratio) and 95% CI (95% confidence interval). Model 1: adjusted for age and sex; Model 2: adjusted by age, sex, calories and fiber. *p* < 0.05 was considered as significant.

The consumption of cheese was associated with lower glucose level in model 1 (OR: 0.73, 95%-CI: 0.55–0.96, *p* = 0.023) and in model 2 (OR: 0.74, 95%-CI: 0.56–0.98, *p* = 0.036) (Table 4). Also, two portions of cheeses/day reduced the risk of having high random blood glucose levels by approximately 80% (Figure 1). However, no association was observed from milk, skimmed yogurt, whole yogurt or curd cheese intake (*p* > 0.05) (Table 4). There was a negative association between dairy products with BMI in model 1 (OR: 0.79, 95%-CI: 0.68–0.93, *p* = 0.004) and model 2 (OR: 0.79, 95%-CI: 0.67–0.92, *p* = 0.004). Furthermore, cheese and dairy products was inversely associated with random blood glucose in model 1 (OR: 0.86, 95%-CI: 0.74–0.99, *p* = 0.042) (Table 4). 

### 3.5. Association between Meat and Egg Intake with Anthropometric Measurements and Blood Glucose 

No associations were observed between meat, such as beef, pork, poultry, fish, and eggs with anthropometric indicators and random blood glucose (*p* > 0.05) (Table 5). 

## 4. Discussion

In the present study, we found that a high-protein diet was associated with lower BMI, WC, TSFT, and random blood glucose. The consumption of dairy products was inversely associated with BMI and random blood glucose. In addition, two portions of cheese/day were inversely associated with random blood glucose. Besides, the intake of beef, pork, poultry, fish, and eggs were not associated with any anthropometric indicators of adiposity or random blood glucose.

Evidence suggests that high-protein diets promote greater weight loss [18]. In this sense, Soenen et al. [19] showed that normal and high-protein diets were able to reduce adiposity. Additionally, protein intakes higher than the current RDA (∼1.2–1.6 g/(kg·day) can be effective to improve appetite control and weight management compared with standard protein diets [2,20,21].

Protein is the macronutrient that is more satiating than carbohydrates and fats [2,4]. Proteins are known to induce satiety and decrease the energy intake, which are fundamental conditions for weight loss [2,4,18]. The satiety effect of protein consumption can be explained by the increase in plasma amino acid concentration in the post-absorption phase combined with secretion of satiety hormones such as cholecystokinin (CCK) and glucagon-like peptide (GLP-1). Another important fact that potentiates weight loss is the thermogenic effect of proteins [8].

In the present study, it was observed that people who consumed a high-protein diet had lower blood glucose levels. Dietary proteins have an insulinotropic effect, which is associated with the composition of amino acids, in particular, BCAA since it improves pancreatic β-cell function besides increasing the sensitivity and secretion of insulin [8,22].

Among the protein groups of our study, cheese and dairy products consumption were associated with lower BMI and random blood glucose. In a systematic review and meta-analysis of randomized controlled clinical trials, Abargouei et al. [23] found that inclusion of dairy products in weight loss energy-restricted diets help to reduce adipose tissue and WC, thereby preserving lean body mass compared with the conventional weight loss diets. On the other hand, Szilagyi. [24] suggested in a narrative review that evaluating the impact of dairy on obesity may be affected by incorporating the effects of lactase genetics. However, it would be important to evaluate the role of dairy separately in either lactase non persistent or lactase persistent populations.

The beneficial effects of dairy consumption may be associated with milk peptides such as glycomacropeptides that stimulate the production of CKK by I cells of small intestine which can modulate satiety [25,26]; to the calcium that decreases the absorption of fatty acids [8,27] and to the effect of improving glucose homeostasis probably through the stimulation of incretin hormones, which increase the insulin release and improve insulin sensitivity [28]. Furthermore, a meta-analysis conducted by Gijsbers et al. [29] suggested a possible role for dairy foods (200 g/day) in the prevention of DM2.

In the present study, two portions of cheeses/day decreased the risk to present altered blood sugar levels by approximately 80%. Similar results was found by Aune et al. [30], who concluded that dairy products consumption, including cheese, was inversely associated with DM2. The likely benefits of fermented dairy products, such as cheese, are related to their positive effects on the intestinal microbiota [31]. In addition, dairy proteins (i.e., casein and whey) stimulates the release of satiety hormones such as CCK and insulin, probably mediated by the faster serum absorption of BCAA and an improvement in glucose homeostasis in DM2 [8,32].

No significant associations were found between the consumption of plant-based protein, the anthropometric parameters of adiposity and glucose. These data differ from those found by Tonstad, Malik and Haddad [33], which verified that the participants with high intake of legumes reduced body weight compared to those with a low-carb diet. However, the result was possibly due to the amount of fiber, rather than the protein contained in these foods, which provided an increase in satiety and greater weight loss. The high content of fiber and polyphenols in the grains contribute to glycemic control, besides having antioxidant activities that help reduce the development of DM2. However, in our study, the amount of fiber consumed seems have not influenced the results, since we adjusted the analysis by this dietary component. 

Our study has some limitations. Although we evaluated only a habitual food recall, limiting the control of seasonality of fruit and vegetable intake, the results found in the present study have an important impact on the food consumption of the population. This evaluation method is considered to be a validated method to evaluate the consumption of the population and has also been used in other studies that evaluated the food consumption of the Brazilian population [34,35]. Mixed macronutrients and the content of sugar in diet were not evaluated in the recall. The self-reported dietary data and under-reporting of energy intake may also be considered limitations of our study [36]. The random blood glucose has not been done on an empty stomach, so the levels of this metabolite can vary greatly. Therefore, we used the reference values for random blood glucose to obtain reliable results [14]. Despite the large variation of age, the statistical analysis was adjusted by this variable reducing the influence on the results. We also highlighted that when we analyzed the consumption of protein sources, all types of milk and cheese were grouped and not separated according to their fat content, which may have influenced the results, but did not invalidate them.

## 5. Conclusions

In conclusion, a high-protein diet containing dairy foods, including cheeses was associated with lower BMI and random blood glucose.

## Figures and Tables

**Table 1:** Demographic profile anthropometric measurements, blood glucose concentrations, food intake of volunteers according to protein consumption.

Variables	Low-protein <0.8 g/kg *N* = 130	Normoprotein 0.8–1.2 g/kg *N* = 130	High-protein >1.2 g/kg *N* = 158	*p*
Sociodemographic				
Age (years)	50.35 ± 16.42A	46.09 ± 14.62AB	40.01 ± 16.91B	<0.001 *^,¥^
Adults (%)	85 (26.90)	100 (31.65)	131 (41.46)	0.002 *
Elderly (%)	45 (44.12)	30 (29.41)	27 (26.47)	
Gender (*n*)				
Men (%)	62 (31.63)	52 (26.53)	82 (41.84)	0.129
Women (%)	68 (30.63)	78 (35.14)	76 (34.23)	
Regular physical activity (*n*)				
Yes (%)	46 (36.38)	48 (36.92)	45 (28.48)	0.257
No (%)	84 (64.62)	82 (63.08)	113 (71.52)	
Smoking (*n*)				
Yes (%)	13 (39.39)	9 (27.27)	11 (33.34)	0.563
No (%)	117 (30.39)	121 (31.43)	147 (38.18)	
Alcohol consumption (*n*)				
Yes (%)	56 (27.59)	67 (33.00)	80 (39.41)	0.317
No (%)	74 (34.42)	63 (29.30)	78 (36.28)	
Anthropometric Measurements				
BMI (kg/m^2^)	27.57 ± 4.43A	26.82 ± 4.70AB	24.26 ± 3.91B	<0.001 *^,¥^
Normal	55 (25.82)	58 (27.23)	100 (46.95)	<0.001*
Overweight	75 (36.59)	72 (35.12)	58 (28.29)	
TSFT (mm)	25.35 ± 9.11A	25.07 ± 8.35AB	20.35 ± 8.08B	<0.001 *^,¥^
WC (cm)	96.19 ± 10.95A	93.41 ± 11.12AB	87.98 ± 11.69B	<0.001 *
Glucose (mg/dL)	110.14 ± 41.89A	106.06 ± 30.53AB	101.55 ± 32.37B	0.007 *^,¥^
Food intake				
Calorie (kcal)	1310.05 ± 539.01A	1782.45 ± 569.93B	2408.76 ± 832.39C	<0.001 *^,¥^
Carbohydrate (%)	50.48 ± 12.79A	45.34 ± 12.08B	39.00 ± 11.36C	<0.001 *^,¥^
Lipids (%)	13.64 ± 4.49A	17.51 ± 6.31B	21.26 ± 6.08C	<0.001 *^,¥^
Protein (%)	15.09 ± 4.74A	15.97 ± 4.54A	17.25 ± 4.05B	<0.001 *^,¥^
Protein (g/kg)	0.56 ± 0.18A	0.98 ± 0.11B	1.80 ± 0.49C	<0.001 *
Leucine (g)	1.36 ± 0.99A	2.24 ± 1.68B	3.83 ± 3.83C	<0.001 *^,¥^
Fiber (g)	15.69 ± 10.83A	19.34 ± 12.83B	22.65 ± 12.17B	<0.001 *^,¥^
Protein (g/kg)				
Adults	0.56 ± 0.17A	0.98 ± 0.11B	1.79 ± 0.49C	<0.001 *^,¥^
Elderly	0.56 ± 0.18A	0.99 ± 0.12B	1.83 ± 0.51C	<0.001 *^,¥^

BMI: Body Mass Index; TSFT: triceps skinfold thickness; WC: Waist circumference. *p*-value obtained by one-way ANOVA (¥ or Kruskall-Wallis) test for continuous variables and chi-square for categorical variables. * *p* < 0.05 was considered statistically different. The different capital letters represent differences in the post-hoc Bonferroni test.

**Table 2:** Association (Odds ratio) between ingestion of legumes with anthropometric measurements and blood glucose concentrations.

Variables	Legumes	*p*
BMI (kg/m^2^)	OR (CI 95%)	
Normal (adults <25 or elderly <27)	1	
Overweight (adults ≥25 or elderly ≥27)		
Crude	1.05 (0.89–1.26)	0.533
Model 1	1.02 (0.85–1.22)	0.828
Model 2	1.03 (0.83–1.29)	0.769
TSFT (Percentile)		
Normal (≥percentile 10 <percentile 90)	1	
High (≥percentile 90)		
Crude	1.12 (0.92–1.35)	0.248
Model 1	1.01 (0.82–1.23)	0.938
Model 2	0.99 (0.78–1.26)	0.964
WC (cm)		
Normal (Women <80 or Men <94)	1	
High (Women ≥80 or Men ≥94)		
Crude	0.92 (0.77–1.11)	0.417
Model 1	1.00 (0.82–1.23)	0.967
Model 2	1.08 (0.84–1.40)	0.548
Glucose (mg/dL)		
Normal (<200)	1	
High (≥200)		
Crude	0.92 (0.74–1.14)	0.455
Model 1	1.15 (0.95–1.39)	0.152
Model 2	1.16 (0.92–1.46)	0.209

BMI: body mass index; TSFT: triceps skinfold thickness; WC: Waist circumference. Logistic regression analysis with OR (Odds Ratio) and 95% CI (95% confidence interval). Model 1: adjusted for age and sex; Model 2: adjusted by age, sex, calories and fiber.

**Table 3:** Association (Odds ratio) between total protein intake with anthropometric measurements and blood glucose concentrations.

Variables	Total Protein Intake	*p*
BMI (kg/m^2^)		
Normal (adults <25 or elderly <27)	1	
Overweight (adults ≥25 or elderly ≥27)		
Crude	0.45 (0.32–0.64)	<0.001
Model 1	0.47 (0.33–0.67)	<0.001
Model 2	0.26 (0.16–0.44)	<0.001
TSFT (Percentile)		
Normal (≥percentile 10 <percentile 90)	1	
High (≥percentile 90)		
Crude	0.55 (0.37–0.81)	0.003
Model 1	0.52 (0.34–0.79)	0.002
Model 2	0.27 (0.15–0.51)	<0.001
WC (cm)		
Normal (Women <80 or Men <94)	1	
High (Women ≥80 or Men ≥94)		
Crude	0.36 (0.25–0.51)	<0.001
Model 1	0.44 (0.30–0.65)	<0.001
Model 2	0.25 (0.14–0.43)	<0.001
Glucose (mg/dL)		
Normal (<200)	1	
High (≥200)		
Crude	0.52 (0.37–0.74)	<0.001
Model 1	0.60 (0.42–0.86)	0.006
Model 2	0.56 (0.34–0.91)	0.019

BMI: body mass index; TSFT: triceps skinfold thickness; WC: Waist circumference. Logistic regression analysis with OR (Odds Ratio) and 95% CI (95% confidence interval). Model 1: adjusted for age and sex; Model 2: adjusted by age, sex, calories and fiber. *p* < 0.05 was considered as significant.

**Table 4:** Association (Odds ratio) between dairy intake with anthropometric measurements and blood glucose concentrations.

Variables	Milk	*p*	Skimmed Yogurt	*p*	Whole Yogurt	*p*	Cheese	*p*	Curd Cheese	*p*	Dairy Products	*p*
BMI (kg/m^2^)	OR (CI 95%)		OR (CI 95%)		OR (CI 95%)		OR (CI 95%)		OR (CI 95%)		OR (CI 95%)	
Normal	1		1		1		1		1		1	
Overweight												
Crude	0.83 (0.67–1.04)	0.107	0.64 (0.20–2.09)	0.463	0.60 (0.35–1.00)	0.051	0.80 (0.62–1.03)	0.088	0.64 (0.12–3.22)	0.585	0.80 (0.67–0.92)	0.003
Model 1	0.83 (0.67–1.04)	0.113	0.68 (0.21–2.21)	0.521	0.63 (0.37–1.06)	0.083	0.78 (0.60–1.01)	0.062	0.68 (0.13–3.39)	0.635	0.79 (0.68–0.93)	0.004
Model 2	0.84 (0.67–1.04)	0.118	0.68 (0.21–2.21)	0.522	0.63 (0.37–1.07)	0.086	0.78 (0.61–1.01)	0.065	0.68 (0.14–3.45)	0.647	0.79 (0.67–0.92)	0.004
TSFT (Percentile)												
Normal	1		1		1		1		1		1	
High												
Crude	0.88 (0.67–1.14)	0.330	0.85 (0.23–2.95)	0.805	0.97 (0.61–1.54)	0.906	1.13 (0.88–1.46)	0.344	0.43 (0.43–4.29)	0.471	1.00 (0.86–1.17)	0.993
Model 1	0.91 (0.71–1.18)	0.497	0.91 (0.89–0.24)	0.887	1.14 (0.70–1.84)	0.602	1.09 (0.83–1.42)	0.543	0.40 (0.04–3.87)	0.428	1.01 (0.87–1.19)	0.821
Model 2	0.91 (0.71–1.18)	0.488	0.90 (0.23–3.49)	0.886	1.14 (0.70–1.85)	0.593	1.09 (0.83–1.42)	0.542	0.40 (0.04–3.85)	0.426	1.01 (0.87–1.20)	0.823
WC (cm)												
Normal	1		1		1		1		1		1	
High												
Crude	0.90 (0.72–1.12)	0.333	0.89 (0.30–2.67)	0.842	0.84 (0.55–1.28)	0.424	0.85 (0.66–1.08)	0.191	0.76 (0.15–3.74)	0.738	0.89 (0.77–1.02)	0.105
Model 1	0.85 (0.67–1.07)	0.160	1.03 (0.25–4.19)	0.966	0.84 (0.54–1.32)	0.459	0.86 (0.66–1.17)	0.259	1.42 (0.27–7.41)	0.681	0.87 (0.75–1.01)	0.078
Model 2	0.84 (0.67–1.07)	0.167	1.03 (0.25–4.20)	0.969	0.83 (0.53–1.31)	0.430	0.86 (0.66–1.12)	0.254	1.44 (0.27–7.61)	0.665	0.86 (0.74–1.01)	0.073
Glucose (mg/dL)												
Normal	1		1		1		1		1		1	
High												
Crude	0.92 (0.74–1.14)	0.455	1.00 (0.35–2.83)	0.995	0.75 (0.47–1.18)	0.212	0.76 (0.58–1.00)	0.053	0.57 (0.11–3.02)	0.508	0.86 (0.74–1.00)	0.052
Model 1	0.91 (0.74–1.14)	0.427	1.12 (0.39–3.23)	0.827	0.80 (0.49–1.30)	0.369	0.73 (0.55–0.96)	0.023	0.69 (0.13–3.63)	0.658	0.86 (0.74–0.99)	0.042
Model 2	0.93 (0.74–1.16)	0.521	1.13 (0.39–3.25)	0.816	0.83 (0.51–1.37)	0.478	0.74 (0.56–0.98)	0.036	0.75 (0.14–4.00)	0.737	0.87 (0.75–1.01)	0.077

BMI: body mass index; TSFT: triceps skinfold thickness; WC: Waist circumference. Logistic regression analysis with OR (Odds Ratio) and 95% CI (95% confidence interval). Model 1: adjusted for age and sex; Model 2: adjusted by age, sex, calories and fiber. Cut-off points BMI (Normal: Adults <25 or elderly <27 and Overweight: Adults ≥25 or elderly ≥27 kg/m^2^) TSFT (Normal: ≥percentile 10 <percentile 90 and High: ≥percentile 90), WC (Normal: Women <80 cm and Men <94 cm and High: Women ≥80 cm and Men ≥94 cm) and Glucose (Normal: <200 mg/dL and High: ≥200 mg/dL). *p* < 0.05 was considered as significant.

**Table 5:** Association (Odds ratio) between meat and egg intake with anthropometric measurements and blood glucose concentrations.

Variables	Beef	*p*	Pork	*p*	Poultry	*p*	Fish	*p*	Eggs	*p*
BMI (kg/m^2^)	OR (CI 95%)		OR (CI 95%)		OR (CI 95%)		OR (CI 95%)		OR (CI 95%)	
Normal	1		1		1		1		1	
Overweight										
Crude	1.05 (0.96–1.15)	0.319	1.43 (0.80–2.56)	0.224	0.94 (0.79–1.12)	0.483	1.11 (0.86–1.45)	0.423	1.06 (0.92–1.24)	0.403
Model 1	1.04 (0.94–1.14)	0.467	1.43 (0.79–2.59)	0.235	0.96 (0.81–1.15)	0.674	1.11 (0.85–1.44)	0.450	1.06 (0.90–1.23)	0.491
Model 2	1.04 (0.94–1.15)	0.388	1.47 (0.81–2.68)	0.202	0.96 (0.81–1.15)	0.697	1.12 (0.85–1.46)	0.417	1.06 (0.91–1.23)	0.481
TSFT (Percentile)										
Normal	1		1		1		1		1	
High										
Crude	1.05 (0.95–1.16)	0.338	1.52 (0.88–2.64)	0.136	0.89 (0.73–1.09)	0.275	1.03 (0.78–1.36)	0.849	1.02 (0.87–1.19)	0.837
Model 1	1.00 (0.90–1.10)	1.000	1.48 (0.81–2.69)	0.199	0.88 (0.72–1.09)	0.257	1.05 (0.78–1.41)	0.748	0.97 (0.82–1.14)	0.700
Model 2	1.00 (0.89–1.11)	0.987	1.50 (0.82–2.77)	0.190	0.88 (0.71–1.09)	0.249	1.05 (0.78–1.42)	0.731	0.97 (0.82–1.14)	0.711
WC (cm)										
Normal	1		1		1		1		1	
High										
Crude	1.00 (0.90–1.10)	0.960	1.78 (0.76–4.13)	0.184	0.82 (0.68–0.98)	0.031	1.23 (0.86–1.74)	0250	0.93 (0.79–108)	0.338
Model 1	1.08 (0.96–1.21)	0.221	2.18 (0.86–5.50)	0.100	0.90 (0.74–1.11)	0.351	1.17 (0.81–1.70)	0.409	0.97 (0.82–1.16)	0.766
Model 2	1.08 (0.96–1.22)	0.189	2.27 (0.89–5.74)	0.084	0.91 (0.75–1.11)	0.367	1.16 (0.79–1.69)	0.446	0.97 (0.81–1.15)	0.711
Glucose (mg/dL)										
Normal	1		1		1		1		1	
High										
Crude	0.99 (0.90–1.08)	0.786	1.50 (0.82–2.73)	0.186	0.86 (0.72–1.04)	0.120	0.98 (0.75–1.28)	0.891	0.88 (0.75–1.03)	0.118
Model 1	0.97 (0.88–1.07)	0.591	1.56 (0.83–2.92)	0.169	0.91 (0.75–1.10)	0.330	0.95 (0.72–1.25)	0.707	0.86 (0.73–1.01)	0.074
Model 2	1.00 (0.90–1.11)	0.970	1.75 (0.93–3.28)	0.081	0.92 (0.76–1.11)	0.400	1.00 (0.75–1.32)	0.996	0.87 (0.74–1.02)	0.096

BMI: body mass index; TSFT: triceps skinfold thickness; WC: Waist circumference. Logistic regression analysis with OR (Odds Ratio) and 95% CI (95% confidence interval). Model 1: adjusted for age and sex; Model 2: adjusted by age, sex, calories and fiber. Cut-off points BMI (Normal: Adults <25 or elderly <27 and Overweight: Adults ≥25 or elderly ≥27 kg/m^2^) TSFT (Normal: ≥percentile 10 <percentile 90 and High: ≥percentile 90), WC (Normal: Women <80 cm and Men <94 cm and High: Women ≥80 cm and Men ≥94 cm) and Glucose (Normal: <200 mg/dL and High: ≥200 mg/dL). *p* < 0.05 was considered as significant.
